# Impact of seasons on stroke-related depression, mediated by vitamin D status

**DOI:** 10.1186/s12888-018-1944-z

**Published:** 2018-11-08

**Authors:** Yingying Gu, Xiaoqian Luan, Wenwei Ren, Lin Zhu, Jincai He

**Affiliations:** 1 Department of Psychiatry, the First Affiliated Hospital of Wenzhou Medical University, Wenzhou, 325000 Zhejiang Province China; 2 Department of Neurology, the First Affiliated Hospital of Wenzhou Medical University, Wenzhou, 325000 Zhejiang Province China; 3 Department of Pediatrics, the First Affiliated Hospital of Wenzhou Medical University, Wenzhou, 325000 Zhejiang Province China

**Keywords:** Season, Depression, Stroke, Vitamin D, Biomarker

## Abstract

**Background:**

This study aimed to describe the seasonal variation of depression prevalence among stroke patients at 1 month and to explore whether vitamin D plays a role in the association between seasons and post-stroke depression (PSD).

**Methods:**

Data were collected from 402 acute stroke patients. Seasons were stratified by summertime (June to November) and wintertime (December to May) based on vitamin D status. The impact of seasons on PSD was assessed via binary logistic regression, with summertime considered the referent category. The mediating effect was used to evaluate whether vitamin D plays a role in the association between seasons and PSD.

**Results:**

The prevalence of PSD was significantly higher in the wintertime group than in the summertime group (*P* = 0.003). The serum vitamin D level was lower in wintertime than in summertime (*P* < 0.001). Lower vitamin D levels were associated with higher HAMD scores (*P* < 0.001). In the multivariate analysis, patients in the wintertime group had a higher prevalence of PSD compared with those in the summertime group across all binary logistic regression models after adjusting for potential confounders. When serum vitamin D was added to the above model, there was no association between seasons and PSD (*P* = 0.056). Vitamin D was independently associated with PSD (OR 0.95, 95% CI 0.935–0.966, *P* < 0.001).

**Conclusion:**

There was a clear seasonal variation in depression prevalence among stroke patients. Vitamin D status plays a critical mediating role in the relationship between season and post-stroke depression.

## Introduction

In China, with a large population of 1.4 billion people, the stroke mortality rate is almost 1.6 million each year, and stroke has become the primary cause of death and adult disability [1]. In addition, China annual new stroke cases is approximately 2.5 million and stroke survivors is almost 7.5 million [2]. Among the complications of stroke, post-stroke depression (PSD) has high clinical relevance. Recently, a meta-analysis of 43 researches that included approximately 2 million stroke survivors suggested that 29% of patients developed depression within the first 5 years following the stroke [3]. PSD aggravated stroke-related adverse events in the form of higher mortality, greater functional disability, raised the risk of recurrent stroke, and lower the quality of life [4], and in the end, with worse rehabilitation outcomes. Although psychological, social and biological factors in the mechanism of depression have been explored [5], the potential pathophysiology of PSD is still uncertain and several diverse mechanisms may be relevant to the development of PSD.

Vitamin D is an essential neurosteroid hormone in human body that may be related to the development of depression. Several studies have been performed in humans demonstrated that vitamin D receptors and vitamin D-activating enzymes existed intracerebral structures such as the hippocampus, the prefrontal cortex, and the amygdala [6, 7]. In addition, vitamin D plays an important role in many brain processes including brain development, regulation of neurotrophic factors, neuroplasticity, neuroprotection, and neuroimmunomodulation [8], which suggesting that vitamin D might be related to the development of depression. Increasing evidence suggests that, low serum levels of 1,25-dihydroxyvitamin D had an association with the increased risk of depression in non-stroke patients [9–12]. Compared with placebo, several studies demonstrated that vitamin D supplementation improved depressive symptom in clinical patients [13, 14]. Nevertheless, in other intervention studies, no significant effect of vitamin D supplementation, compared with placebo, was found on changes in depressive symptom scores [15, 16]. Meanwhile, several studies examining the relationship between vitamin D and depression have been performed in stroke patients, and the results from these studies confirmed an inverse relationship between serum vitamin D levels and PSD [17–19].

Increasing evidence suggests that, there was a seasonal variation in depression. One study demonstrated that depressive symptoms during autumn and winter were more common than during the summer among senior high school students in Swedish [20]. Similarly, a study with 11054 general population suggested that seasonality is associated with depression [21]. In addition, another study suggested that the prevalence of postpartum depression (PD) in winter time was higher than other seasons in Taiwan [22]. Depressive symptoms are common during wintertime when vitamin D levels may be reduced due to inadequate ultraviolet B radiation leading to decreased vitamin D synthesis in the skin [23, 24]. Since serum vitamin D levels were usually higher in summertime than wintertime, if reduced vitamin D levels were a reason of PSD, then depression was supposed to be more common in wintertime when compared to summertime in stroke survivors. This study aimed to describe the seasonal variation of depression prevalence among stroke patients at 1 month post stroke and explore whether serum vitamin D levels plays an important role in the association between seasons and PSD.

## Methods

### Study design

This was an observational study. The study was performed from October 2013 to May 2015 and was made up of acute stroke patients aged 18-80 years at Department of Neurology, the First Affiliated Hospital of Wenzhou Medical University, Zhejiang, China (27°-28° degrees latitude). All patients had been hospitalized within 7 days of stroke first symptom, and a clinical diagnosis of acute stroke was confirmed by computerized tomography (CT) reports or magnetic resonance imaging (MRI). Patients with their serum vitamin D levels measured were included. The exclusion criteria were the following: (1) patients with a malignant tumor, a pre-stroke diagnosis dementia or a severe cognitive impairment, visual or auditory impairment or aphasia making them unable to participate in the clinical psychological tests, (2) patients with a history of mental disorders (especially depression, anxiety and other psychiatric disorders), (3) patients with another significant neurology diseases (e.g., Parkinson’s disease), (4) patients suffered from osteoporosis or had used vitamin D and/or calcium supplementation previously.

This study was approved by the Medical Ethics Committee of the First Affiliated Hospital of Wenzhou Medical University and was performed in line with the principles of the Declaration of Helsinki. We have obtained written informed consent from participants or their closest relatives.

### Data collection and measures

At admission, the health examination included demographic data (age and gender) and the records of weight, height, and blood pressure, and the characteristic data such as years of education, history of conventional vascular risk factors, family history of mental disorders, and smoking and drinking history. BMI was calculated based on the information of height and weight.

At 1 month after the stroke, a psychological evaluation was performed by a trained psychiatrist/neurologist who was unaware of the patients’ other information. The 17-item Hamilton Rating Scale for Depression (HAMD) was used to assess the presence of depressive symptoms at the same time [25]. The diagnosis of depression was in line with the Diagnostic and Statistical Manual of Mental Disorders, fourth edition (DSM-IV) criteria on the basis of the depressive symptoms (HAMD score > 7). The severity of stroke was evaluated at admission with the National Institutes of Health Stroke Scale (NIHSS) by trained neurologists [26].

A fasting blood sample was drawn for analyses of serum vitamin D levels within 24 h of admission. The measurement of serum vitamin D levels was carried out using a competitive protein-binding assay at the First Affiliated Hospital of Wenzhou Medical University’s laboratory. The intraassay coefficient of variation was 7%–10%. The season of the blood sampling was grouped as spring (March to May), summer (June to August), autumn (September to November) and winter (December to February). Vitamin D serum levels were higher during autumn 55.88 (37.28-68.61) nmol/L and summer 64.29 (47.39-81.00) nmol/L and were lower during spring 41.87 (28.34-58.39) nmol/L and winter 47.29 (33.94-60.31) nmol/L. According to the Osteoporosis Committee of China Gerontological Society for vitamin D and bone health in adult Chinese, the definition (25(OH) D < 30 nmol/L indicating deficiency, 30 to 49.9 nmol/L indicating insufficiency, and ≥50 nmol/L indicating sufficiency) [27], the extended seasons were categorized as summertime (June to November) and wintertime (December to May) based on vitamin D status [28].

### Statistical analysis

Analyses were conducted to describe and to compare demographic and clinical characteristics at baseline between patients with and without depression. In addition, all analyses were conducted between seasons stratified by summertime and wintertime. The Kolmogorov-Smirnov test was used for a normal distribution. Serum vitamin D levels were not normal distribution. Non-normal distributed variables were expressed as medians (quartiles) and analyzed with the Mann-Whitney test. Normal distributed variables were presented as the mean ± standard deviation (SD) and analyzed with Student's t-test. Categorical variables were expressed as percentages and numbers and analyzed with the chi-squared test. The correlation between vitamin D and depression was examined by Spearman correlation coefficients. The impact of seasons on PSD was performed via binary logistic regression, with summertime considered the referent category. The mediating effect was used to evaluate whether vitamin D played a role in the association between seasons and PSD. The variables associated with PSD and seasons at baseline with *P* values < 0.1 were taken into account as potential confounders. Age, BMI, gender were also selected. Analyses were adjusted for gender and age in Model 1. Analyses were further adjusted for BMI, history of diabetes mellitus, history of hyperlipidemia, history of stroke, history of coronary heart disease, alcohol consumption, and active smoking status in Model 2. In Model 3, analyses were additionally adjusted with stroke subtypes, and the NIHSS score at admission was taken into account. Model 4 included model 3 and added vitamin D. The results were presented as adjusted odds ratios (OR) along with the corresponding 95% confidence intervals (CI). All the analyses were conducted in SPSS 20.0 (IBM, SPSS, and Chicago, IL). For all analyses, a *P*-value < 0.05 based on a two-sided test was considered statistically significant.

## Results

### Baseline characteristics of study samples

The study enrolled 551 acute stroke patients at admission. This analysis was limited to stroke survivors who were followed up at 1 month. There were 149 such patients excluded from the study: 143 patients had missing data and 6 patients passed away. Thus, the final sample comprised 402 acute stroke patients. When compared to the 149 patients excluded from the study, there were no statistically significant differences in gender, age, BMI and NIHSS scores.

In the study sample, the mean age of the stroke patients was 62.41± 10.24 years. 270 (67.2%) of the included patients were male and 132 (32.8%) were female. The median serum vitamin D level was 49.52 (32.71-64.14) nmol/L with no significant difference between women and men (50.16 (32.70-63.86) nmol/L vs 46.98 (31.63-66.01) nmol/L, *P* = 0.67). The median HAND score was 4 (2-8).

### Vitamin D

**Fig. 1:**
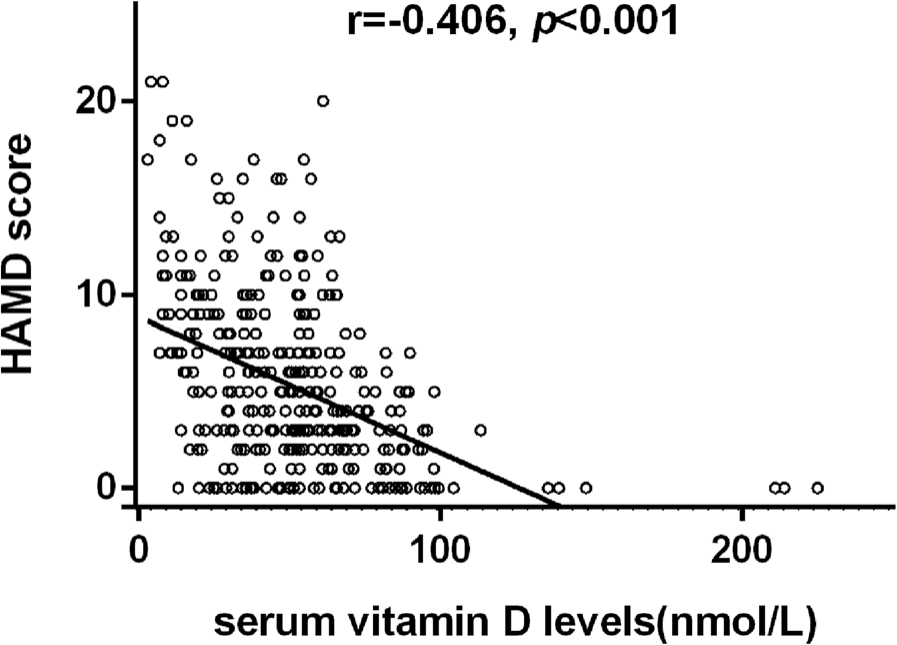
The correlation between serum vitamin D levels and HAMD scores, r [spearman] = − 0.406, *p* < 0.001

As shown in Table 1, patients in the wintertime group showed lower serum vitamin D levels than those in the summertime group (46.98 (30.90-57.88) nmol/L vs 52.44 (46.04-68.11) nmol/L, *P* < 0.001). Serum vitamin D levels were statistically significant higher in the non-PSD patients than in the PSD patients (51.06(36.44-63.95) nmol/L vs 43.80(30.40-55.88) nmol/L, *P* = 0.011). A higher HAMD score was significantly associated with lower serum vitamin D levels (*r* = -0.406, *P* < 0.001) Fig. 1.

**Table 1:** Characteristics of the study population (*n* = 402) according to stratification of season and post-stroke depression

	Stratification of season			Post-stroke depression		
	Wintertime (*n* = 265)	Summertime (*n* = 137)	*P*-value	Non-PSD (*n* = 295)	PSD (*n* = 107)	*P-*value
Demographic characteristics						
Age (years), mean ± SD	62.47 ± 10.22	62.29 ± 10.31	0.871	62.85 ± 10.29	61.18 ± 10.04	0.149
Female, n (percent)	86 (32.5)	46 (33.6)	0.82	94 (31.9)	38 (35.5)	0.491
BMI (kgm^−2^), mean ± SD	24.00 ± 3.28	23.97 ± 3.29	0.899	23.84 ± 3.18	24.42 ± 2.94	0.1
Education (years), median (IQR)	5 (0–7)	4.5 (1–7)	0.889	4.5 (0–7)	5 (0–7)	0.862
Vascular risk factors (%)						
Hypertension	192 (72.7)	101 (73.7)	0.831	211 (71.8)	82 (76.6)	0.331
Diabetes mellitus	55 (20.9)	46 (33.6)	0.006	76 (25.9)	25 (23.4)	0.6
Hyperlipidemia	18 (6.9)	6 (4.4)	0.316	14 (4.8)	10 (9.4)	0.086
Coronary heart disease	19 (7.3)	4 (2.9)	0.076	20 (6.9)	3 (2.9)	0.136
History of stroke	23 (8.8)	20 (14.6)	0.075	32 (10.9)	11 (10.4)	0.877
Active smokers	82 (31.5)	37 (27.6)	0.421	93 (32.2)	26 (24.8)	0.156
Alcohol consumption	94 (37.3)	46 (35.9)	0.794	111 (39.8)	29 (28.7)	0.048
Systolic blood pressure (mmHg), mean ± SD	153.68 ± 21.38	158.45 ± 24.54	0.055	154.78 ± 22.91	156.76 ± 21.72	0.439
Diastolic blood pressure (mmHg), mean ± SD	82.96 ± 13.16	85.34 ± 13.03	0.085	83.40 ± 13.34	84.79 ± 12.63	0.149
Stroke subtype			0.629			0.001
Hemorrhagic stroke	47 (17.7)	27 (19.7)		43 (14.6)	31 (29.0)	
Ischemia stroke	218 (82.3)	110 (80.3)		252 (85.4)	76 (71.0)	
NIHSS score	3 (1–4)	3 (1–4)	0.909	2 (1–4)	3 (2–6)	< 0.001
Vitamin D (nmol/L), median (IQR)	46.98 (30.90–57.88)	52.44 (46.04–68.11)	< 0.001	51.06 (36.44–63.95)	43.80 (30.40–55.88)	0.011
HAMD at 1 month, median (IQR)	5 (2–9)	3 (2–6.5)	0.005	3 (1–5)	10 (9–13)	< 0.001

Values are shown as number (percentage) or as medians (IQR) and means (±SD)

*PSD* post-stroke depression, *SD* standard deviation, *IQR* interquartile range, *BMI* body mass index, *NIHSS* National Institutes of Health Stroke Scale, *HAMD* Hamilton Rating Scale for Depression

### Season and post-stroke depression

Table 1 summarized the characteristics of the study population according to stratification of season and for patients with or without depression. Compared to the patients without depression, characteristics associated with depression in the univariate analyses were active alcohol consumption, hemorrhagic stroke, higher initial stroke severity and lower vitamin D levels. There were no statistically significant differences in demographic data. In our study, 26.6% of acute stroke patients (107 patients) were diagnosed as PSD. The prevalent rate of PSD was significantly higher in the wintertime group than in the summertime group (31.3% vs 17.5%, *P* =0.003) (Table 2). In addition, patients in the wintertime group had higher HAMD scores than those in the summertime group (5 (2-9) vs 3 (2-6.5), *P* < 0.0001). There were no statistically significant differences in other demographic characteristics and clinical data with regard to stratification of the seasons (Table 1).

**Table 2:** Prevalent of post-stroke depression by season

	Summertime (*n* = 137)	Wintertime (*n* = 265)	*P*-value
All stroke patients (*n* = 402)			0.003
Stroke with depression (*n* = 107)	24 (17.5)	83 (31.3)	
Stroke without depression (*n* = 295)	113 (82.5)	182 (68.7)	

Table 3 showed the results of binary logistic regression analysis for the association between PSD and stratification of the seasons. The prevalent rate of PSD was significantly higher in the wintertime group compared with those in the summertime group in an unadjusted model (OR: 2.14; 95% CI: 1.28-3.58, *P* = 0.003). Furthermore, patients in the wintertime group had a higher prevalent rate of PSD compared with those in the summertime group across all binary logistic regression models after adjusted potential confounders. In Model 1, after adjustment for age and gender, and summertime taken as the reference, wintertime was independently related to the prevalence of PSD (OR:2.17; 95% CI:1.29-3.63). After full adjustment numerous underlying confounding factors, the relationship between season and PSD was further strengthened with an OR (95% CI) of 2.37(1.29-4.34). While when serum vitamin D was added in above described model 3, there was no association between seasons and PSD (*P* = 0.056), and vitamin D was independently related to PSD (OR 0.95, 95% CI 0.935-0.966, *P* < 0.001).

**Table 3:** Odds ratio (95% confidence interval) for 1-month post-stroke depression by season

	Post-stroke depression	
	OR (95% CI)	*P*-value
Unadjusted Model	2.14 (1.28–3.58)	0.003
Model 1	2.15 (1.28–3.60)	0.003
Model 2	2.23 (1.28–3.88)	0.005
Model 3	2.60 (1.43–4.72)	0.002
Model 4	1.87 (0.98–3.56)	0.064

Take summertime as the referent category. Model 1 included age and gender. Model 2 included model 1 and added BMI, history of hyperlipidemia, history of Diabetes mellitus, history of Coronary heart disease, history of stroke, smoke, alcohol consumption. Model 3 included model 2 and added admission NIHSS score, and stroke subtype. Model 4 included model 3 and added vitamin D

*OR* odds ratios, *CI* confidence intervals

## Discussion

To the best of my knowledge, this is the first study investigating the impact of seasons on stroke-related depression. The present study suggested that there was a higher prevalent rate of PSD in the wintertime, when serum vitamin D levels were expected to be lower. Full adjustment for all the potential confounders related to seasons and PSD at admission had minimal effect on the results. In addition, lower serum vitamin D levels were associated with a higher HAMD score. Our results suggest that seasons were associated with the prevalence of PSD and that the association was mediated by vitamin D status. Low vitamin D levels induced by seasonal variation play a vital role in the presence of PSD.

In this study, we found that 26.6% patients (107 patients) were diagnosed as PSD at 1 month post stroke, and the prevalent rate of PSD was significantly higher in the wintertime group compared to those in the summertime group, which was in line with previous researches. A few studies have demonstrated seasonal mood changes to be related to various mental diseases. For example, a recent study consisting of 202 individuals suggested that the highest prevalence depressive symptoms in bipolar I disorder was observed around the winter solstice and the lowest frequency in the summer [29]. In another study, a group of 2107 women with PD were analyzed, and the results demonstrated that the prevalent rate of PD for winter deliveries was higher than other seasons in Taiwan [22]. In addition, Cobb et al found that there exists statistically significant peak in depressive symptoms from the winter solstice to the spring equinox in unipolar major depressive disorder [30]. Moreover, a recent study found that the highest proportion of major depressive episodes occurred in winter and the lowest proportions occurred in summer; seasonal variation was clearly evident [31]. Regarding the abovementioned studies, depressive symptoms are more common during wintertime than summertime, our findings are consistent with previous results.

In addition, we demonstrated that patients in the summertime group were more possibly to have higher serum vitamin D levels than those in the wintertime group, similar to previously reported results [32–34], because of enhanced sun/UVB irradiation dose which was an efficient way to acquire adequate vitamin D levels [35]. Sun exposure and estimated UV radiation significantly predicted serum vitamin D levels, and result in higher serum vitamin D levels during summer months. Moreover, serum vitamin D levels were significantly higher in non-PSD patients compared with in PSD patients, and a higher HAMD score was significantly related to lower vitamin D levels, which was in line with the results of previous researches. Previous studies have founded a strong association between depression and vitamin D in non-stroke patients [10, 11, 36–38], whereas others show no association [39, 40]. However, there have been few studies conducted in stroke patients. One study found that vitamin D deficiency was associated with depression symptoms in stroke patients [17], and others found a relationship between increased depression prevalent rate and lower serum vitamin D levels in stroke patients [18, 19].

A number of plausible pathogenesis underlying the relationship between low vitamin D level and PSD could be speculated about. As a unique neurosteroid hormone that has acrossed the blood–brain barrier, vitamin D plays an important role in many brain processes such as brain development, neuroplasticity, and regulation of neurotrophic factors [8], which may contribute to the pathophysiology of depression. Second, another probable pathogenesis between depression and low vitamin D levels is by regulating neurotransmitters such as norepinephrine, dopamine, and serotonin, which involved in the pathogenesis of depression, which are target neurotransmitters for antidepressants treatment [41]. The impact of vitamin D on neurotransmission may contribute to depression [42–44]. Moreover, vitamin D affects inflammatory pathways (promoting anti-inflammatory pathways by VDR-mediated gene transcription and down-regulating autoimmune pathways producing proinflammatory cytokines) [45, 46] that in turn to play a role in neuroprotection. In acute stroke, clinical studies and experimental have found that the ischemic brain tissue reacts to ischemic injury with a prolonged and acute inflammatory process, characterized by production of proinflammatory mediators, rapid activation of resident cells, and infiltration of all kinds of inflammatory cells [47]. A number of studies confirmed proof for the vital role of inflammation in the pathophysiologic mechanisms of depression by neurotransmitter systems, regulating neuroplasticity [48], and HPA axis activation [49]. In acute stroke survivors, lower vitamin D may contribute to PSD due to modulating the relationship between depression and inflammatory responses. Overall, the pathophysiology between lower vitamin D and PSD includes inflammation, neurogenesis in response to ischemia and low vitamin D, and alterations in serotonergic, noradrenergic, and dopaminergic pathways that lead to changes in amine levels.

Our study has some important strengths, this is the first study investigating the impact of seasons on stroke-related depression. Furthermore, our study included a measure of actual serum vitamin D levels, a diagnoses of depression, and a large sample size (402 patients). However, there are several limitations of this study that need to be addressed. First, participants with depression or other severe aphasia and severe cognitive impairment were excluded, which are likely to be underrepresented in the present study sample. Second, serum vitamin D levels was only tested at admission; therefore, our study have no data about how vitamin D was changed during the follow up. Third, we failed to acquire information on weather or temperature, physical functioning and participation in sports activities for the amount of time spent outdoors and during sunlight exposure, which may have a significant impact on vitamin D levels. In addition, information on whether patients with thyroid diseases were not recorded. Furthermore, only one point (1 month) was for the evaluation of PSD and a longer time point (e.g., 3 month or 6 month) would strengthen the manuscript. Finally, this study was conducted in a purely Chinese ethnic background and patients aged from18 to 80 years.

## Conclusion

In summary, we found that PSD is more common in wintertime when serum vitamin D levels are expected to be lower compared with those in summertime, and a higher HAMD score were related to lower serum vitamin D levels; these data support the suggestion that the association is mediated by vitamin D status. Our results suggest that seasons are associated with the prevalence of PSD and the association is mediated by vitamin D status. Future studies should be performed in stroke patients followed through numerous seasons, meanwhile to explore the role of other confounders that influence by season, include daylight length, temperature, and physical activity.

## Abbreviations

BMI: Body mass index
CI: Confidence intervals
CT: Computerized tomography
DSM-IV: The Diagnostic and Statistical Manual of Mental Disorders, fourth edition
HAMD: Hamilton Rating Scale for Depression
MRI: Magnetic resonance imaging
NIHSS: National Institutes of Health Stroke Scale
OR: Odds ratios
PSD: Post-stroke depression
SD: Standard deviation

## References

[1] Liu L, Wang D, Wong KS, Wang Y (2011). Stroke and stroke care in China: huge burden, significant workload, and a national priority. Stroke.

[2] Johnston SC, Mendis S, Mathers CD (2009). Global variation in stroke burden and mortality: estimates from monitoring, surveillance, and modelling. Lancet Neurol.

[3] Ayerbe L, Ayis S, Wolfe CD, Rudd AG (2013). Natural history, predictors and outcomes of depression after stroke: systematic review and meta-analysis. Br J Psychiatry.

[4] Ayerbe L, Ayis S, Crichton S, Wolfe CD, Rudd AG (2014). The long-term outcomes of depression up to 10 years after stroke; the South London Stroke Register. J Neurol Neurosurg Psychiatry.

[5] Krishnan V, Nestler EJ (2010). Linking molecules to mood: new insight into the biology of depression. Am J Psychiatry.

[6] Zehnder D, Bland R, Williams MC, McNinch RW, Howie AJ, Stewart PM, Hewison M (2001). Extrarenal expression of 25-hydroxyvitamin d (3)-1 alpha-hydroxylase. J Clin Endocrinol Metab.

[7] Eyles DW, Smith S, Kinobe R, Hewison M, McGrath JJ (2005). Distribution of the vitamin D receptor and 1 alpha-hydroxylase in human brain. J Chem Neuroanat.

[8] Fernandes de Abreu DA, Eyles D, Feron F, Vitamin D (2009). a neuro-immunomodulator: implications for neurodegenerative and autoimmune diseases. Psychoneuroendocrinology.

[9] Johansson P, Alehagen U, van der Wal MH, Svensson E, Jaarsma T (2016). Vitamin D levels and depressive symptoms in patients with chronic heart failure. Int J Cardiol.

[10] Milaneschi Y, Hoogendijk W, Lips P, Heijboer AC, Schoevers R, van Hemert AM, Beekman AT, Smit JH, Penninx BW (2014). The association between low vitamin D and depressive disorders. Mol Psychiatry.

[11] Anglin RE, Samaan Z, Walter SD, McDonald SD (2013). Vitamin D deficiency and depression in adults: systematic review and meta-analysis. Br J Psychiatry.

[12] Brouwer-Brolsma EM, Dhonukshe-Rutten RA, van Wijngaarden JP, van der Zwaluw NL, Sohl E, In't Veld PH, van Dijk SC, Swart KM, Enneman AW, Ham AC (2016). Low vitamin D status is associated with more depressive symptoms in Dutch older adults. Eur J Nutr.

[13] Sepehrmanesh Z, Kolahdooz F, Abedi F, Mazroii N, Assarian A, Asemi Z, Esmaillzadeh A (2016). Vitamin D Supplementation Affects the Beck Depression Inventory, Insulin Resistance, and Biomarkers of Oxidative Stress in Patients with Major Depressive Disorder: A Randomized, Controlled Clinical Trial. J Nutr.

[14] Stokes CS, Grunhage F, Baus C, Volmer DA, Wagenpfeil S, Riemenschneider M, Lammert F (2016). Vitamin D supplementation reduces depressive symptoms in patients with chronic liver disease. Clin Nutr.

[15] Bertone-Johnson ER, Powers SI, Spangler L, Larson J, Michael YL, Millen AE, Bueche MN, Salmoirago-Blotcher E, Wassertheil-Smoller S, Brunner RL (2012). Vitamin D supplementation and depression in the women's health initiative calcium and vitamin D trial. Am J Epidemiol.

[16] Kjaergaard M, Waterloo K, Wang CE, Almas B, Figenschau Y, Hutchinson MS, Svartberg J, Jorde R (2012). Effect of vitamin D supplement on depression scores in people with low levels of serum 25-hydroxyvitamin D: nested case-control study and randomised clinical trial. Br J Psychiatry.

[17] Kim SH, Seok H, Kim DS (2016). Relationship Between Serum Vitamin D Levels and Symptoms of Depression in Stroke Patients. Ann Rehabil Med.

[18] Yue W, Xiang L, Zhang YJ, Ji Y, Li X (2014). Association of serum 25-hydroxyvitamin D with symptoms of depression after 6 months in stroke patients. Neurochem Res.

[19] Han B, Lyu Y, Sun H, Wei Y, He J (2015). Low serum levels of vitamin D are associated with post-stroke depression. Eur J Neurol.

[20] Rastad C, Ulfberg J, Sjoden PO (2006). High prevalence of self-reported depressive mood during the winter season among Swedish senior high school students. J Am Acad Child Adolesc Psychiatry.

[21] Oyane NM, Bjelland I, Pallesen S, Holsten F, Bjorvatn B (2008). Seasonality is associated with anxiety and depression: the Hordaland health study. J Affect Disord.

[22] Yang SN, Shen LJ, Ping T, Wang YC, Chien CW (2011). The delivery mode and seasonal variation are associated with the development of postpartum depression. J Affect Disord.

[23] Engelsen O, Brustad M, Aksnes L, Lund E (2005). Daily duration of vitamin D synthesis in human skin with relation to latitude, total ozone, altitude, ground cover, aerosols and cloud thickness. Photochem Photobiol.

[24] Tsiaras WG, Weinstock MA (2011). Factors influencing vitamin D status. Acta Derm Venereol.

[25] Hamilton M (1960). A rating scale for depression. J Neurol Neurosurg Psychiatry.

[26] Brott T, Adams HPJ, Olinger CP, Marler JR, Barsan WG, Biller J, Spilker J, Holleran R, Eberle R, Hertzberg V (1989). Measurements of acute cerebral infarction: a clinical examination scale. Stroke.

[27] Liao XP, Zhang ZL, Zhang HH, Zhu HM, Zhou JL, Huang QR, Wang ZX, Wang L, Liu ZH (2014). Application Guideline for Vitamin D and Bone Health in Adult Chinese (2014 Standard Edition). Chin J Osteopor.

[28] Meyer HE, Robsahm TE, Bjorge T, Brustad M, Blomhoff R (2013). Vitamin D, season, and risk of prostate cancer: a nested case-control study within Norwegian health studies. Am J Clin Nutr.

[29] Akhter A, Fiedorowicz JG, Zhang T, Potash JB, Cavanaugh J, Solomon DA, Coryell WH (2013). Seasonal variation of manic and depressive symptoms in bipolar disorder. Bipolar Disord.

[30] Cobb BS, Coryell WH, Cavanaugh J, Keller M, Solomon DA, Endicott J, Potash JB, Fiedorowicz JG (2014). Seasonal variation of depressive symptoms in unipolar major depressive disorder. Compr Psychiatry.

[31] Patten SB, Williams JV, Lavorato DH, Bulloch AG, Fiest KM, Wang JL, Sajobi TT (2017). Seasonal variation in major depressive episode prevalence in Canada. Epidemiol Psychiatr Sci.

[32] Park KY, Chung PW, Kim YB, Moon HS, Suh BC, Won YS, Kim JM, Youn YC, Kwon OS (2015). Serum Vitamin D Status as a Predictor of Prognosis in Patients with Acute Ischemic Stroke. Cerebrovasc Dis.

[33] Perna L, Felix JF, Breitling LP, Haug U, Raum E, Burwinkel B, Schottker B, Brenner H (2013). Genetic variations in the vitamin D binding protein and season-specific levels of vitamin D among older adults. Epidemiology.

[34] Klenk J, Rapp K, Denkinger MD, Nagel G, Nikolaus T, Peter R, Koenig W, Bohm BO, Rothenbacher D (2013). Seasonality of vitamin D status in older people in Southern Germany: implications for assessment. Age Ageing.

[35] Howe WR, Dellavalle R (2007). Vitamin D deficiency. N Engl J Med.

[36] Lee DM, Tajar A, O'Neill TW, O'Connor DB, Bartfai G, Boonen S, Bouillon R, Casanueva FF, Finn JD, Forti G (2011). Lower vitamin D levels are associated with depression among community-dwelling European men. J Psychopharmacol.

[37] Milaneschi Y, Shardell M, Corsi AM, Vazzana R, Bandinelli S, Guralnik JM, Ferrucci L (2010). Serum 25-hydroxyvitamin D and depressive symptoms in older women and men. J Clin Endocrinol Metab.

[38] Polak MA, Houghton LA, Reeder AI, Harper MJ, Conner TS (2014). Serum 25-hydroxyvitamin D concentrations and depressive symptoms among young adult men and women. Nutrients.

[39] Chan R, Chan D, Woo J, Ohlsson C, Mellstrom D, Kwok T, Leung P (2011). Association between serum 25-hydroxyvitamin D and psychological health in older Chinese men in a cohort study. J Affect Disord.

[40] Pan A, Lu L, Franco OH, Yu Z, Li H, Lin X (2009). Association between depressive symptoms and 25-hydroxyvitamin D in middle-aged and elderly Chinese. J Affect Disord.

[41] Tekes K, Gyenge M, Folyovich A, Csaba G (2009). Influence of neonatal vitamin A or vitamin D treatment on the concentration of biogenic amines and their metabolites in the adult rat brain. Horm Metab Res.

[42] Brown J, Bianco JI, McGrath JJ, Eyles DW (2003). 1,25-dihydroxyvitamin D3 induces nerve growth factor, promotes neurite outgrowth and inhibits mitosis in embryonic rat hippocampal neurons. Neurosci Lett.

[43] Cass WA, Smith MP, Peters LE (2006). Calcitriol protects against the dopamine- and serotonin-depleting effects of neurotoxic doses of methamphetamine. Ann N Y Acad Sci.

[44] Patrick RP, Ames BN (2015). Vitamin D and the omega-3 fatty acids control serotonin synthesis and action, part 2: relevance for ADHD, bipolar disorder, schizophrenia, and impulsive behavior. FASEB J.

[45] Howren MB, Lamkin DM, Suls J (2009). Associations of depression with C-reactive protein, IL-1, and IL-6: a meta-analysis. Psychosom Med.

[46] Shang YX, Ding WQ, Qiu HY, Zhu FP, Yan SZ, Wang XL (2014). Association of depression with inflammation in hospitalized patients of myocardial infarction. Pak J Med Sci.

[47] Jin R, Yang G, Li G (2010). Inflammatory mechanisms in ischemic stroke: role of inflammatory cells. J Leukoc Biol.

[48] Fuchs E, Czeh B, Kole MH, Michaelis T, Lucassen PJ (2004). Alterations of neuroplasticity in depression: the hippocampus and beyond. Eur Neuropsychopharmacol.

[49] Himmerich H, Binder EB, Kunzel HE, Schuld A, Lucae S, Uhr M, Pollmacher T, Holsboer F, Ising M (2006). Successful antidepressant therapy restores the disturbed interplay between TNF-alpha system and HPA axis. Biol Psychiatry.

